# Vaccination coverage, barriers and vaccine hesitancy in children up to 24 months old: a population survey in a state capital in the Western Amazon

**DOI:** 10.1590/S2237-96222024v33e20231295.especial2.en

**Published:** 2024-11-01

**Authors:** Thaiane Rodrigues de Oliveira Macedo, Maria Fernanda de Sousa Oliveira Borges, Ilce Ferreira da Silva, Ana Paula França, José Cássio de Moraes, ICV 2020 Group, Adriana Ilha da Silva, Alberto Novaes Ramos, Ana Paula França, Andrea de Nazaré Marvão Oliveira, Antonio Fernando Boing, Carla Magda Allan Santos Domingues, Consuelo Silva de Oliveira, Ethel Leonor Noia Maciel, Ione Aquemi Guibu, Isabelle Ribeiro Barbosa Mirabal, Jaqueline Caracas Barbosa, Jaqueline Costa Lima, José Cássio de Moraes, Karin Regina Luhm, Karlla Antonieta Amorim Caetano, Luisa Helena de Oliveira Lima, Maria Bernadete de Cerqueira Antunes, Maria da Gloria Teixeira, Maria Denise de Castro Teixeira, Maria Fernanda de Sousa Oliveira Borges, Rejane Christine de Sousa Queiroz, Ricardo Queiroz Gurgel, Rita Barradas Barata, Roberta Nogueira Calandrini de Azevedo, Sandra Maria do Valle Leone de Oliveira, Sheila Araújo Teles, Silvana Granado Nogueira da Gama, Sotero Serrate Mengue, Taynãna César Simões, Valdir Nascimento, Wildo Navegantes de Araújo

**Affiliations:** 1 Universidade Federal do Acre, Programa de Pós-Graduação em Saúde Coletiva, Rio Branco, AC, Brazil; 2 Escola Nacional de Saúde Pública Sergio Arouca, Fundação Oswaldo Cruz, Rio de Janeiro, RJ, Brazil; 3 Faculdade de Ciências Médicas da Santa Casa de São Paulo, Departamento de Saúde Coletiva, São Paulo, SP, Brazil; Universidade Federal do Espírito Santo, Vitória, ES, Brazil; Universidade Federal do Ceará, Departamento de Saúde Comunitária, Fortaleza, CE, Brazil; Faculdade Ciências Médicas Santa Casa de São Paulo, São Paulo, SP, Brazil; Secretaria de Estado da Saúde do Amapá, Macapá, AP, Brazil; Universidade Federal de Santa Catarina, SC, Brazil; Organização Pan-Americana da Saúde, Brasília, DF, Brazil; Instituto Evandro Chagas, Belém, PA, Brazil; Faculdade de Ciências Médicas Santa Casa de São Paulo, Departamento de Saúde Coletiva, São Paulo, SP, Brazil; Universidade Federal do Rio Grande do Norte, Natal, RN, Brazil; Universidade Federal do Ceará, Departamento de Saúde Comunitária, Fortaleza, CE, Brazil; Universidade Federal de Mato Grosso, Cuiabá, MT, Brazil; Universidade Federal do Paraná, Curitiba, PR, Brazil; Universidade Federal de Goiás, Goiânia, GO, Brazil; Universidade Federal do Piauí, Teresina, PI, Brazil; Universidade de Pernambuco, Faculdade de Ciências Médicas, Pernambuco, PE, Brazil; Instituto de Saúde Coletiva, Universidade Federal da Bahia, Salvador, BA, Brazil; Secretaria de Estado da Saúde de Alagoas, Maceió, AL, Brazil; Universidade Federal do Acre, Rio Branco, AC, Brazil; Universidade Federal do Maranhão, Departamento de Saúde Pública, São Luís, MA, Brazil; Universidade Federal de Sergipe, Aracaju, SE, Brazil; Secretaria Municipal de Saúde, Boa Vista, RR, Brazil; Fundação Oswaldo Cruz, Mato Grosso do Sul, Campo Grande, MS, Brazil; Fundação Oswaldo Cruz, Escola Nacional de Saúde Pública Sergio Arouca, Rio de Janeiro, RJ, Brazil; Universidade Federal do Rio Grande do Sul, Porto Alegre, RS, Brazil; Fundação Oswaldo Cruz, Instituto de Pesquisa René Rachou, Belo Horizonte, MG, Brazil; Secretaria de Desenvolvimento Ambiental de Rondônia, Porto Velho, RO, Brazil; Universidade de Brasília, Brasília, DF, Brazil

**Keywords:** Programas de Inmunización, Cobertura de Vacunación, Vacilación Ante las Vacunas, Vacunas, Encuestas Epidemiológicas, Immunization Programs, Vaccination Coverage, Vaccination Hesitation, Vaccines, Health Surveys

## Abstract

**Objective:**

To estimate vaccination coverage, identify barriers and hesitancy to vaccinating children up to 24 months, born between 2017-2018, living in the urban area of Rio Branco, Acre, Brazil.

**Methods:**

Population survey carried out from 2020 to 2021, which assessed sociodemographic characteristics and vaccination status among children.

**Results:**

Among 451 included children, vaccination coverage was below 80%. Meningococcal C vaccine had the lowest coverage for administered doses (76.3%; 95%CI 70.5;81.3) and doses on time (27.4%; 95%CI 23.1;32.1). The statements “vaccines cause serious adverse reactions” (26.4%; 95%CI 18.1;36.8) and “you don’t need vaccination for diseases that no longer exist” (22%; 95%CI 15.7;29.8) were the most frequent regarding vaccination hesitancy. Lack of vaccines was the main barrier to care (86.6%; 95%CI 71.8;94.3).

**Conclusion:**

Vaccination coverage in children born in 2017-2018 was below the target recommended for the full schedule of administered doses, both valid and timely administered.

## Study contributions

**Main results:**

Coverage levels below 80%. Main barriers: access difficulties and lack of vaccines. Believing that events supposedly attributable to vaccination are serious was the most frequent statement justifying vaccination hesitancy.

**Implications for services:**

It is necessary to avoid loss of vaccination opportunities, guaranteeing vaccines and qualified professionals in health centers, in addition to providing guidance to the community on the vaccination schedule and its intervals.

**Perspectives:**

Prospective and qualitative studies help to assess associated factors and understand barriers and vaccination hesitancy; evaluating the cost-effectiveness of programs aimed at the role of health professionals is essential.

## RESUMEN

**Objetivo:**

Estimar la cobertura vacunal, identificar barreras y reticencias a la vacunación en niños de hasta 24 meses, nacidos en 2017-2018, residentes en el área urbana de Rio Branco-Acre.

**Métodos:**

Encuesta poblacional realizada de 2020-2021. Se evaluaron características sociodemográficas y estado de vacunación.

**Resultados:**

En los 451 niños estudiados la cobertura vacunal por esquema fue inferior al 80%. La cobertura más baja para dosis aplicadas (76,3%; IC_95_% 70,5;81,3) y dosis oportunas (27,4%; IC_95_% 23,1;32,1) fue para meningococo C Las afirmaciones “las vacunas causan reacciones adversas graves” (26,4%; IC_95_% 18,1;36,8) y “no hay necesidad de vacuna para enfermedades que ya no existen” (22%; IC_95_% 15,7;29,8) fueron las más frecuentes en relación con la reticencia a las vacunas. (22%; IC_95_% 15,7;29,8) fueron las más frecuentes respecto a la renuencia a vacunarse. La falta de vacuna fue la principal barrera para la atención (86,6%; IC_95_% 71,8;94,3).

**Conclusión:**

La cobertura vacunal en niños nacidos en 2017-2018 estuvo por debajo de la meta recomendada en los esquemas completos de dosis aplicadas, válidos y oportunos.

## INTRODUCTION

In order to control indicators of vaccine-preventable diseases, the World Health Organization (WHO) recommends a percentage of vaccination coverage greater than 95%, which is a relevant public health measure.^1^ However, the downward trend and inequalities in coverage are evident at regional and global levels, and high-income countries have higher coverage when compared to low-income countries.^2^ Between 2019 and 2021, a study that assessed global and regional inequality in six WHO regions (Africa, America, Eastern Mediterranean, Europe, Southeast Asia and Western Pacific) showed that, of these regions, Europe had the highest coverage for a group of 11 vaccines on the childhood schedule, while Africa had the lowest coverage.^3^ Another study showed that less than half of Latin American countries achieved 90% coverage.^4^

The challenges to improving vaccination coverage are not restricted to economic issues alone. The increased notion of risk of events supposedly attributable to vaccination or immunization (ESAVI) and the decreased understanding of disease risk also become obstacles, resulting in vaccination hesitancy, even in high-income countries.^5^

The Brazilian National Immunization Program (*Programa Nacional de Imunizaç*ões - PNI) has stood out worldwide for coordinating immunization actions universally and free of charge.^6^ However, the decline in vaccination coverage has generated concern about the possibility of the return of diseases.^7^

An ecological study carried out in Brazil, with data from the PNI Information System (SI-PNI), between 2011 and 2020, found significant decreasing trends in the vaccination coverage of immunobiologicals: tuberculosis vaccine, with a negative average annual percentage change of 3.58 %; 5-in-1 (diphtheria, tetanus, pertussis, hepatitis B and *Haemophilus influenzae B*), 4.10%; polio virus vaccine, 2.76%; and MMR (measles, mumps and rubella), 2.56%. The North showed the biggest drop in coverage of these vaccines among the Brazilian regions. Lower socioeconomic development, difficulties in vaccine availability, and geographic and access barriers contributed to disparities.^8^

Based on SI-PNI estimates for vaccination coverage, dropout rate and population size, the largest concentration of municipalities with high and very high risk of transmission of vaccine-preventable diseases in the country is found in the states of Acre, Amazonas, Amapá, Pará, Piauí and Roraima.^9^ In 2021, the state of Acre, located in the Western Amazon, had the second lowest coverage for the first dose of the MMR vaccine (60.2%) and the fifth lowest coverage for the third dose against polio virus (61.8%).^10^

Although vaccination coverage in Brazil has been predominantly estimated based on data recorded in health centers, some objections are raised regarding recording errors and estimates of the target population. To overcome such problems, carrying out population surveys, with the aim of gaining a better estimation of vaccination coverage, measuring inequalities and investigating barriers to vaccination, is an important initiative.^11^

Therefore, this study aims to estimate vaccination coverage and identify barriers to vaccination and vaccination hesitancy among children up to 24 months old, born between 2017 and 2018, and living in the urban area of Rio Branco-AC, Brazil. 

## METHODS

This is a population-based household survey, part of the 2020 National Vaccination Coverage Survey (*Inquérito Nacional de Cobertura Vacinal 2020* - INCV 2020), carried out from December 2020 to May 2021, with children born in 2017 and 2018, residing in the urban area of Rio Branco-AC, Brazil, the methodology of which has been detailed in another article.^11^

Rio Branco is the capital of the state of Acre. In 2020, its estimated resident population was 413,418, 7.53% of whom were children between 0 and 4 years old, and the birth rate was 13.89 live births per 1,000 inhabitants. The target population of this research were the 12,955 live births in 2017 and 2018 (6,460 and 6,495, respectively), to mothers living in the municipality, according to data from the Live Birth Information System (*Sistema de Informação sobre Nascidos Vivos* - SINASC).

Sampling was carried out in three stages. The first corresponded to socioeconomic stratification by census tracts (A – high, B – medium high, C – medium low and D – low), classified according to the 2010 Demographic Census. In the second stage, census tract clusters were formed, based on the estimated number of live births held on the SINASC (2017-2018), four clusters being drawn from each socioeconomic stratum. In the third stage, a search was carried out for children living in the clusters. Considering 70% expected prevalence of vaccinated children, 95% confidence interval (95%CI) and design effect of 1.4, the estimated sample size was 452 children in the municipality. 

The data were collected by a specialized company. Identification of children was carried out using SINASC data (2017-2018). In situations that prevented the interview from being carried out (addresses not found; children who did not live there; refusal; and guardian was not at home after two attempts), a replacement was made with another child born in 2017 or 2018 who lived in the same cluster. The interviewers used a standardized questionnaire (via electronic device), answered by the child’s mother or guardian. Information about vaccines was obtained from the child’s vaccination card. 

Vaccination coverage was calculated by dividing the number of children who received doses of vaccines on the schedule by the total number of children in the sample, multiplied by 100, taking into account the schedule up to 24 months old.^13^ The schedules were divided into vaccines that should be administered from 0-12 and from 12-24 months of life, with the combination of doses administered from 0-24 months of life subsequently being analyzed. Coverage was calculated for each schedule, considering three indicators: administered, valid and on-time doses.

The 0-12 month schedule includes one dose of tuberculosis vaccine ‒ Bacillus Calmette-Guérin (BCG) ‒, one dose against hepatitis B, three doses of 5-in-1, three doses against polio virus, two doses and a booster of pneumococcal vaccine, two doses of oral human rotavirus vaccine, two doses and a booster of meningococcal C vaccine, first dose of MMR and one dose against yellow fever. The 12-24 month schedule includes one dose of hepatitis A vaccine, a second dose of MMR, a booster against poliomyelitis, a DTP (diphtheria, tetanus and pertussis) booster and one dose against varicella. The two previous schedules are combined together to form the 0-24 months vaccination schedule.^13^

When calculating the vaccination coverage of doses administered, the numerator was the sum of children who had the vaccines administered for each schedule (0-12, 12-24 and 0-24 months), without taking into account the season and intervals; in the case of valid and on-time doses, the numerator was the sum of those who had vaccines for each schedule administered at specific intervals.^12^ The denominator, for all three doses, was the total number of children in the sample.

Vaccination hesitancy followed the WHO 3Cs model, which is based on three main categories: confidence, complacency and convenience.^13^ Barriers to vaccination were related to personal or health service reasons that made vaccination difficult or prevented it. 

A descriptive analysis was carried out using relative frequencies of sociodemographic and economic variables: maternal age at childbirth (≤ 20 years; 21-34 years; ≥ 35 years), maternal race/skin color (White; non-White), maternal schooling (years of study: 0-8; 9-12; 13-15; ≥ 16); maternal marital status (has a partner: yes; no); number of children alive (1; 2-3; ≥ 4); maternal paid job (yes; no); child’s sex (male; female); child’s race/skin color (White; non-White); child’s birth order (first; second/third; fourth or more); *Bolsa Família* benefit (yes; no); and monthly family income (≤ BRL 1000; BRL 1001-BRL 3000; > BRL 3000). 

Information was collected regarding vaccination hesitancy and the main barriers to vaccination, namely: “vaccines are important for children’s health and collective protection”; “children need to be vaccinated against diseases that no longer exist”; “vaccines cause serious adverse reactions”; “confidence in vaccines distributed by the government”; “difficulties in getting the child vaccinated”; “the child was not vaccinated, even though it had been taken to the vaccination center”; and “stopped vaccinating their child due to their own decision”. 

In all analyses, the values corresponding to the effect arising from the use of cluster sampling in multiple stages were considered, enabling unbiased estimation of the parameters of interest in the population.^11^ The estimates considered the weights corresponding to the different population sizes in each stratum. The analyses were performed using SPSS version 21.0. 

The study was approved by the Research Ethics Committee of the *Instituto de Saúde Coletiva da Universidade Federal da Bahia*, as per Opinion No. 3.366.818, on June 4, 2019, and Certificate of Submission for Ethical Appraisal (*Certificado de Apresentação de Apreciação* Ética - CAAE) No. 4306919.5.0000.5030; and by the Research Ethics Committee of the *Irmandade da Santa Casa de São Paulo*, as per Opinion No. 4.380.019, on November 4, 2020, and CAAE No. 39412020.0.0000.5479.

## RESULTS

The study included 451 children, with 0.22% losses. Of the total number of children, 50% (95%CI 42.5;57.5) were male; the majority, non-White (73.8%; 95%CI 67.5;80.5); and were in second/third position in birth order (45.6%; 95%CI 39.4;51.9). Regarding family characteristics, the majority of mothers were between 21 and 34 years old (63.7%; 95%CI 57.2;69.7), were of non-White race/skin color (81.5%; 95%CI 72.3;88.1), with 13 to 15 years of schooling (47.8%; 95%CI 37.2;58.6), had a partner (74.0%; 95%CI 65.7;80.9), had between two and three children (48.6%; 95%CI 39.4;58.0), did not work (57.4%; 95%CI 46.8;67.3 ), belonged to socioeconomic stratum D (41.2%; 95%CI 25.3;59.2), did not receive *Bolsa Família* benefit (63%; 95%CI 54.6;70.8) and had monthly family income between BRL 1001 and BRL 3000 (54.2%; 95%CI 42.9;65.1) (Table 1).

**Table 1:** Characterization of sociodemographic and economic aspects among children up to 24 months old and their mothers, living in Rio Branco, Acre, Brazil, Vaccination Coverage Survey, 2020 (n = 451)

Variables	%	95%CI^a^
**Child’s sex**		
Male	50.0	42.5;57.5
Female	50.0	42.5;57.5
**Child’s race/skin color as reported by its legal guardian**		
White	26.2	19.5;34.3
Non-White	73.8	65.7;80.5
**Child’s birth order**		
First	41.3	33.8;49.2
Second/third	45.6	39.4;51.9
Fourth or more	13.0	9.3;18.1
**Maternal age group at childbirth**		
≤ 20 years	8.5	4.7;14.9
21-34 years	63.7	57.2;69.7
≥ 35 years	27.8	22.6;33.8
**Self-reported maternal race/skin color** ^b^		
White	18.5	11.9;27.7
Non-white	81.5	72.3;88.1
**Maternal schooling (years of study)** ^b^		
0-8 years	12.8	7.1;22.0
9-12 years	10.6	7.1;15.5
13-15 years	47.8	37.2;58.6
16 years or over	28.8	23.1;35.3
**Maternal marital status (has a partner)** ^b^		
Yes	74.0	65.7;80.9
No	26.0	19.1;34.3
**Number of mother’s children alive**		
1	34.6	25.5;44.9
2-3	48.6	39.4;58.0
4 or more	16.8	12.3;22.5
**Maternal paid job** ^b^		
Yes	42.6	32.7;53.2
No	57.4	46.8;67.3
**Socioeconomic stratum**		
A	7.3	4.5;11.7
B	16.4	11.0;23.6
C	35.1	21.1;52.2
D	41.2	25.3;59.2
*Bolsa* *Família* **benefit**		
Yes	37.0	29.2;45.4
No	63.0	54.6;70.8
**Monthly family income** ^b^		
≤ BRL 1000	27.1	18.2;38.4
BRL 1001-BRL 3000	54.2	42.9;65.1
> BRL 3000	18.7	13.3;25.5

a) 95%CI: 95% confidence interval; b) Variable with missing data (< 5%).

With regard to vaccination administration between 0 and 12 months, 78.0% (95%CI 72.2;82.8) had vaccination coverage with doses administered; 58.8% (95%CI 54.0;65.5), with valid doses; and 12.4% (95%CI 8.6;17.7), with on-time doses. Of the vaccines to be administered between 12 and 24 months, 64.2% (95%CI 57.9;70.1) of the children had vaccination coverage with doses administered; 55.5% (95%CI 48.9;61.9), with valid doses; and 9.6% (95%CI 5.6;46.0), with on-time doses. With regard to vaccination between 0 and 24 months, 60.3% (95%CI 53.0;67.2) had vaccination coverage with doses administered; 39.3% (95%CI 35.0;43.8), with valid doses; and 5.4% (95%CI 2.2;12.6), with on-time doses (Table 2).

**Table 2:** Vaccination coverage by schedule and vaccine, valid and on-time second doses administered among children up to 24 months old, living in Rio Branco, Acre, Brazil, Vaccination Coverage Survey, 2020 (n = 451)

Schedule and vaccine	Doses administered % (95%CI)^a^	Valid doses % (95%CI)^a^	On-time doses % (95%CI)^a^
0-12 months vaccination schedule	78.0 (72.2;82.8)	58.8 (54.0;65.5)	12.4 (8.6;17.7)
12-24 months vaccination schedule	64.2 (57.9;70.1)	55.5 (48.9;61.9)	9.6 (5.6;46.0)
0-24 months vaccination schedule	60.3 (53.0;67.2)	39.3 (35.0;43.8)	5.4 (2.2;12.6)
BCG	92.3 (89.0;94.7)	92.3 (89.0;94.7)	89.4 (85.9;92.1)
Hepatitis B	92.9 (89.5;95.2)	92.9 (89.5;95.2)	89.4 (85.9;92.2)
5-in-1 (1^st^ dose)	96.1 (91.8;98.2)	96.0 (91.8;98.1)	74.8 (69.2;79.6)
Polio virus (1^st^ dose)	96.1 (91.8;98.2)	95.9 (91.5;98.0)	83.8 (79.6;87.3)
Pneumococcal (1^st^ dose)	95.1 (91.2;97.3)	94.6 (90.8;96.9)	81.2 (76.3;85.3)
Rotavirus (1^st^ dose)	91.9 (88.7;94.2)	90.3 (87.6;92.5)	76.9 (72.2;81.0)
Meningococcal C (1^st^ dose)	94.8 (90.9;97.1)	94.8 (90.9;97.1)	68.7 (61.6;75.1)
5-in-1 (2^nd^ dose)	95.3 (91.5;97.4)	95.1 (91.4;97.3)	56.7 (50.3;62.9)
Polio virus (2^nd^ dose)	95.4 (91.6;97.5)	95.4 (91.6;97.5)	65.4 (59.1;71.3)
Pneumococcal (2^nd^ dose)	93.6 (90.3;95.9)	93.2 (89.9;95.5)	61.4 (52.7;69.5)
Rotavirus (2^nd^ dose)	86.8 (81.9;90.5)	69.4 (63.1;75.1)	52.9 (46.1;59.5)
Meningococcal C (2^nd^ dose)	91.0 (87.1;93.8)	90.1 (86.1;93.1)	47.8 (41.8;53.8)
5-in-1 (3^rd^ dose)	92.8 (89.3;95.3)	91.7 (88.3;94.1)	37.3 (28.7;46.8)
Yellow fever	89.2 (85.2;92.3)	85.0 (80.4;88.7)	39.1 (34.2;44.2)
Polio virus (3^rd^ dose)	91.2 (86.6;94.2)	90.9 (86.4;94.0)	50.3 (41.2;59.4)
Pneumococcal (booster)	84.1 (79.2;87.9)	82.1 (77.1;86.2)	37.8 (31.1;45.0)
Meningococcal C (booster)	76.3 (70.5;81.3)	73.9 (67.4;79.6)	27.4 (23.1;32.1)
MMR (1^st^ dose)	91.4 (88.1;93.9)	90.5 (87.8;92.7)	40.1 (34.9;45.5)
Hepatitis A	89.1 (85.2;92.1)	88.1 (84.2;91.2)	42.4 (35.7;49.5)
MMR (2^nd^ dose)	78.9 (72.8;83.9)	75.7 (69.7;80.8)	29.8 (22.8;37.9)
Polio virus (1^st^ booster)	84.8 (80.9;87.9)	81.2 (75.9;85.6)	39.2 (31.2;47.8)
DTP (1^st^ booster)	78.2 (72.6;83.0)	78.2 (72.6;83.0)	29.7 (23.0;37.5)
Varicella	80.8 (74.4;85.9)	79.1 (73.0;84.0)	32.0 (24.5;40.6)

a) 95%CI: 95% confidence interval.

When looking at the vaccines in isolation, the lowest coverage for administered doses was found for the meningococcal booster (76.3%; 95%CI 70.5;81.3), followed by the DTP booster (78.2%; 95%CI 72.6;83.0), while the highest coverage was recorded for the 1^st^ dose of 5-in-1 (96.1%; 95%CI 91.8;98.2) and the 1^st^ dose against polio virus (96 .1%; 95%CI 91.8;98.2). The lowest coverage for valid doses was found for the 2^nd^ dose of human rotavirus vaccine (69.4%; 95%CI 63.1;75.1) and the meningococcal C booster (73.9%; 95%CI 67.4 ;79.6); the highest coverage was for the 1^st^ dose of the 5-in-1 vaccine (96.0%; 95%CI 91.8;98.1) and the 1^st^ dose of the polio virus vaccine (95.9%; 95%CI 91.5;98.0). The lowest coverage for on-time doses was found for the meningococcal C booster (27.4%; 95%CI 23.1;32.1), the 1^st^ DTP booster (29.7%; 95%CI 23.0;37 .5) and the 2^nd^ dose of the MMR vaccine (29.8%; 95%CI 22.8;37.9); the highest coverage was for BCG (89.4%; 95%CI 85.9;92.1) and hepatitis B vaccine (89.4%; 95%CI 85.9;92.2) (Table 2).

Regarding vaccination hesitancy, confidence was assessed by asking questions about serious adverse reactions and confidence in vaccines. With regard to complacency, the questions focused on the decision not to vaccinate the child (individuals who answered “yes” spoke about the reasons for the decision); the importance of vaccines for children’s health and collective protection; and the need for vaccines for diseases that no longer exist. Regarding convenience, questions focused on difficulty in taking the child to be vaccinated (individuals who answered “yes” reported what these difficulties were). Children not being vaccinated, even though they had been taken to the vaccination center, also represented a barrier (individuals who answered “yes” reported the reason why vaccination did not happen).

With regard to the data on vaccination hesitancy data, the majority agreed that vaccines are important for children’s health (99.9%; 95%CI 99.5;100.0) and collective protection (99.2%; 95%CI 95.6;99.9). However, 22% (95%CI 15.7;29.8) thought that the child does not need to be vaccinated for diseases that no longer exist, and 26.4% (95%CI 18.1;36.8 ) thought that vaccines cause serious adverse reactions. The majority (94.3%; 95%CI 89.3;97.1) trust vaccines provided by the government. 6.1% (95%CI 3.3;11.0) faced difficulty in taking their child to be vaccinated, 2.7% (95%CI 1.3;5.7) stopped vaccinating their child based on their own decision, and 40.1% (95%CI 33.1;47.6) had taken their child to be vaccinated, but were unsuccessful (Table 3). Regarding barriers to vaccination, among those who reported difficulties in taking their child to be vaccinated (6%), the most frequent were “the health center is a long way away” (77.5%; 95%CI 53.9;91.0), and “lack of transport” (41.5%; 95%CI 21.4;65.0). Regarding reasons for children not having been vaccinated, even though they had been taken to the health center (40.1%), the most common reason was lack of vaccine (86.6%; 95%CI 71.8;94.3) (Table 4 ).

**Table 3:** Characterization of vaccination hesitation among children up to 24 months old, living in Rio Branco, Acre, Brazil, Vaccination Coverage Survey, 2020 (n = 451)

Variables	%	95%CI^a^
**Vaccines are important for children’s health**		
Unfavorable/indifferent	0.1	0.0;0.5
Favorable	99.9	99.5;100.0
**Vaccines are important for collective protection**		
Unfavorable/indifferent	0.8	0.1;4.4
Favorable	99.2	95.6;99.9
**Children need to be vaccinated against diseases that no longer exist**		
Unfavorable/indifferent	22.0	15.7;29.8
Favorable	78.0	70.2;84.3
**Vaccines do not cause serious adverse reactions**		
Unfavorable/indifferent	26.4	18.1;36.8
Favorable	73.6	63.2;81.9
**Confidence in vaccines provided by public services**		
Unfavorable/indifferent	5.7	2.9;10.7
Favorable	94.3	89.3;97.1
**Have you ever had difficulty in vaccinating your child?** ^a^		
Yes	6.1	3.3;11.0
No	93.9	89.0;96.7
**Has your child ever not been vaccinated, despite having been taken to the vaccination center?**		
Yes	40.1	33.1;47.6
No	59.9	52.4;66.9
**Have you ever not vaccinated your child because of your own decision?** ^a^		
Yes	2.7	1.3;5.7
No	97.3	94.3;98.7
**Reasons for having taken the decision not to vaccinate** ^c^		
Fear of reaction to vaccines or reactions that occurred previously	61.4	22.1;89.9
Pandemic	43.0	15.3;75.9
Fear of giving child an injection	38.5	11.3;75.4
Believes that vaccines are bad for health	33.9	14.1;61.5
News stories made them give up	24.8	4.2;71.5
Friend or relative advised not to vaccinate	10.9	1.7;46.1
Doctor advised not to vaccinate	6.1	1.3;24.8
Does not believe in vaccines	2.5	0.3;18.4
Child had a cold	2.3	0.3;16.7

a) 95%CI: 95% confidence interval; b) Variable with missing data (< 5%); c) Total sample size reduced (15); missing data of the type “not applicable”.

**Table 4:** Characterization of barriers to vaccination among children up to 24 months old, living in Rio Branco, Acre, Brazil, Vaccination Coverage Survey, 2020 (n = 451)

Main difficulties in taking child to be vaccinated^a^	%	95%CI^c^
Vaccination center far away from home or work	77.5	53.9;91.0
No means of transport to get to the vaccination center	41.5	21.4;65.0
Lack of time to take the child	24.5	10.6;46.8
Vaccination center opening times inadequate	18.1	6.1;42.6
Has no money for getting to the vaccination center	8.6	2.6;24.4
Boss won’t give time off work	7.7	1.7;28.7
Difficulty moving around	3.7	0.8;15.5
Does not known when to take the child	3.0	0.5;16.9
**Reasons for child not having been vaccinated, despite having been taken to the health center** ^b^		
No vaccine	86.6	71.8;94.3
No health professional	11.3	6.7;18.5
Not the right day for that vaccine	10.0	2.4;33.6
Vaccination room closed	6.3	2.9;13.1
Health professional recommended not administering several vaccines on the same day	5.9	2.0;16.4
Lack of supplies	1.7	0.4;5.9
Child not vaccinated for lack of document	1.0	0.2;4.3
A lot of people in the line and could not wait	0.1	0.0;0.8

a) Total sample size reduced (32); b) Total sample size reduced (170); missing data of the type “not applicable”; c) 95%CI: 95% confidence interval.

## DISCUSSION

The survey of vaccination coverage up to 24 months of age, carried out among children born in 2017 and 2018, living in the urban area of Rio Branco-AC, Brazil, showed vaccination coverage below 80% for the full schedules of administered, valid and on-time doses of vaccines that should be administered from 0 to 12 months, from 12 to 24 months and from 0 to 24 months. Believing that vaccines cause serious adverse reactions and that it is not necessary to be vaccinated against diseases that no longer exist were the most frequent statements related to vaccination hesitancy. Lack of vaccine was the main barrier to health care. 

When assessing vaccination coverage, an important step is to check it according to administered, valid and timely, whereby the latter category makes it possible to note decrease or increase in timely vaccination.^14^ There is evidence that children with full vaccination have 27% greater protection against risk of death, compared to those with overdue vaccination.^15^

In this study, coverage for vaccines administered exactly at the ages recommended by the schedule was low, revealing delay in vaccination. Based on scheduled doses, all vaccines assessed had coverage below 90%, in particular meningococcal C vaccine, which showed low coverage for all doses.

An ecological study described coverage of administered doses of meningococcal C vaccine in children up to 12 months old in the states and regions of Brazil in 2012, two years after the vaccine was included on the national schedule. The North and Northeast regions did not achieve the recommended levels of 95% coverage. In the North, coverage for the first, second dose and booster were, respectively, 89.9%, 84.4% and 67.0%. In the state of Acre, coverage was, respectively, 89.9%, 86.2% and 51.9%.^16^

Nevertheless, a considerable reduction in the number of estimated cases of meningococcal meningitis was seen after its inclusion on the vaccination program (2011, 2012 and 2013) for children under 1 year old (65.7%; 95%CI 44.9;86 .5%) and from 1 to 4 years old (51.8%; 95%CI 33.0;70.6%). In the 5-9 year and 10 years and over age groups, there was a statistically significant reduction in the incidence of the disease in 2013, according to a study that evaluated the impact of vaccination against this disease.^17^

However, in order to control or eliminate vaccine-preventable diseases, achieving high vaccination coverage is not enough; it is necessary to maintain high coverage levels, so as not to compromise the progress made over the years. In this sense, attention must be paid to the control of poliomyelitis and measles, since, in relation to vaccination against these diseases, there are high downward trends in coverage.^18^

In the present study, the second dose against rotavirus also showed low coverage of valid doses. Oral human rotavirus vaccine was introduced in 2006 as part of the Brazilian National Immunization Program. In 2007 it had already led to a 14% reduction in hospitalizations for diarrheal conditions, and an average reduction of 48% in hospitalizations of children under 5 years of age.^19^ Notwithstanding, according to the SI -PNI, in 2022, oral human rotavirus vaccine coverage was 73%, while the target for mass immunity for this vaccine is 90%.^20^

Administration of oral human rotavirus vaccine is subject to limitations, with a very strict age range recommendation. The recommendation is two doses, the first at 2 months (which can be between 1 month and 15 days, and 3 months and 15 days) and the second at 4 months (which can be from 3 months and 15 days to 7 months and 29 days). Children over 4 months and 15 days who have not received any dose will not be able to start the schedule from this age group onwards, as administration outside these deadlines can lead to complications whereby harm outweighs the benefits of the vaccine.^21^

Another important aspect of vaccination coverage relates to analysis of the full schedule, instead of just describing vaccines in isolation, as high coverage of specific vaccines does not ensure high coverage of the full schedule.^14^ Assessment of vaccination coverage from 0-12, 12-24 and 0-24 months enables monitoring the schedule development of the main vaccines that should be administered in early childhood, identifying drops in vaccination coverage, and the most frequent drop period.^22^

In this study, vaccines with multi-dose schedules showed a decrease in subsequent doses percentages, as well as coverage of the 12-24 month schedule being lower than that of the 0-12 month schedule, demonstrating vaccination delay or dropout after the first year of life. In Porto Alegre-RS, from 2015 to 2017, multi-dose vaccines had an average annual loss four times greater than the target set by the WHO, which recommends maximum losses of 5% and 25% for single-dose and multi-dose vaccines, respectively.^23^ In Araraquara-SP, a population-based study to assessing timely vaccination coverage of children aged 12-24 months, born between 1998 and 2013, showed that delays are accentuated from 6 months onwards, being more related to age than to the number of vaccination schedule doses.^24^

Therefore, identifying the reasons that lead a child’s guardian to delay vaccination or decide not to vaccinate their child is of great importance. This delay can be influenced both by the individual difficulties faced by the guardian and also by beliefs or factors related to health services.^25^

A household survey carried out by the Avaaz network and the Brazilian Immunization Society (*Sociedade Brasileira de Imunizações* - SBIm) to assess Brazilians’ perception of vaccines, found that fear of side effects (24.0%) and fear of contracting the disease they were trying to prevent (18.0%) were the most frequent reasons for vaccination hesitation.^26^

However, it is important to highlight that the vaccines provided by the SUS are carefully analyzed, and the frequency of severe ESAVI is considered rare, in contrast to the risk of illness, sequelae and deaths resulting from vaccine-preventable diseases.^27^

The frequency of vaccine-associated paralytic poliomyelitis (VAPP), for example, is between 1 case per 2.4 million doses and 1 case per 13 million doses. Events such as immunoallergenic reactions and anaphylaxis are considered rare (0.1%) or extremely rare (< 0.01%) for vaccines forming part of the infant vaccination schedule.^28^

When we explored the barriers to vaccination reported by those responsible for the children, distance from the health center was the most frequent factor. Large distances between the child’s home and the primary healthcare center are a limiting factor that can lead to vaccination delays or dropout. A survey carried out in the city of Assis Brasil-AC showed that the likelihood of children living in places further away from the health center being vaccinated was lower, considering that the cost of transport to the service was a barrier, especially for lower-income families.^29^

Lack of vaccines and health professionals also represent barriers to improving vaccination coverage. Even if the number of primary healthcare centers increases, optimizing their distribution over the territory, the inefficiency of the logistical process of supplying and assessing vaccines, and the unavailability of qualified professionals could invalidate the efforts undertaken to improve the vaccination status of children living in remote areas. A key factor, in the context of vaccination, is organize the health service flow so that there is no shortage of vaccines, health professionals, and supplies to administer them.^9^

In Cuiabá-MT, lack of vaccine was also the most frequent reason for non-vaccination (50.0%). This is a barrier related to the organization of health services and government management, which can compromise adherence and the opportunity for vaccination.^30^

This was one of the first population-based studies to evaluate vaccination coverage, addressing hesitancy and barriers resulting in non-vaccination in Rio Branco-AC, Brazil. The sample size was a limitation for identifying the reasons given by legal guardians for not vaccinating their child, as it had a high percentage of fields filled in as “not applicable”, considering that only 2.7% of respondents reported having some difficulty vaccinating their child. Therefore, in order not to generate the erroneous idea of probability estimation, these reasons were not explored in depth, being only described in table format.

Although the original study design included sampling with replacement by another child from the same cluster, the losses due to refusal accounted for 0.22%. Thus, despite surveys being subject to selection bias due to refusals to participate, the percentage of losses due to refusals was very low, reducing the likely of selection bias, and ensuring the representativeness of the population in the sample. 

Another limitation of this study refers to information bias (specifically, prevarication bias) regarding the topic of vaccination hesitancy. As it is a sensitive topic, respondents may not feel comfortable expressing their real opinion. This study also did not allow for a longitudinal and prospective approach to enable better assessment of the vaccination coverage indicator in Rio Branco, Acre. However, data relating to vaccines were validated via the children’s vaccination cards, which provide accurate information. 

This study pointed out that vaccination coverage schedules in Rio Branco-AC are below recommended levels, with even lower vaccination percentages after the first year of age. Furthermore, it showed that fear of adverse effects of vaccines was the main factor for vaccination hesitancy, while lack of vaccines and/or health professionals were the main barriers resulting in non-vaccination, despite the child having been taken to the health center. In order to expand vaccination coverage, it is essential for primary care to be strengthened – so that there is no loss of vaccination opportunities due to service management and access reasons, as well as to ensure there are qualified professionalsto perform vaccination, ensure adequate vaccination registration, and provide guidance to the community regarding vaccination and recommended timelines.
