# Supplementary material for: Vaccination coverage, barriers and vaccine hesitancy in children up to 24 months old: a population survey in a state capital in the Western Amazon
Source: Epidemiol Serv Saude. 2024 Nov 1;33(spe2):e20231295. doi: 10.1590/S2237-96222024v33e20231295.especial2.en (PMC11537177; doi:10.1590/S2237-96222024v33e20231295.especial2.en)
Supplement: Translation

# Translation: Cobertura vacinal, barreiras e hesitação vacinal em crianças de até 24 meses: inquérito populacional em uma capital do oeste amazônico

**DOI:** 10.1590/S2237-96222024v33e20231295.especial2.pt

**Authors:** Thaiane Rodrigues de Oliveira Macedo, Maria Fernanda de Sousa Oliveira Borges, Ilce Ferreira da Silva, Ana Paula França, José Cássio de Moraes, Grupo ICV 2020, Adriana Ilha da Silva, Alberto Novaes Ramos, Ana Paula França, Andrea de Nazaré Marvão Oliveira, Antonio Fernando Boing, Carla Magda Allan Santos Domingues, Consuelo Silva de Oliveira, Ethel Leonor Noia Maciel, Ione Aquemi Guibu, Isabelle Ribeiro Barbosa Mirabal, Jaqueline Caracas Barbosa, Jaqueline Costa Lima, José Cássio de Moraes, Karin Regina Luhm, Karlla Antonieta Amorim Caetano, Luisa Helena de Oliveira Lima, Maria Bernadete de Cerqueira Antunes, Maria da Gloria Teixeira, Maria Denise de Castro Teixeira, Maria Fernanda de Sousa Oliveira Borges, Rejane Christine de Sousa Queiroz, Ricardo Queiroz Gurgel, Rita Barradas Barata, Roberta Nogueira Calandrini de Azevedo, Sandra Maria do Valle Leone de Oliveira, Sheila Araújo Teles, Silvana Granado Nogueira da Gama, Sotero Serrate Mengue, Taynãna César Simões, Valdir Nascimento, Wildo Navegantes de Araújo

**Affiliations:** 1 Universidade Federal do Acre, Programa de Pós-Graduação em Saúde Coletiva, Rio Branco, AC, Brasil; 2 Escola Nacional de Saúde Pública Sergio Arouca, Fundação Oswaldo Cruz, Rio de Janeiro, RJ, Brasil; 3 Faculdade de Ciências Médicas da Santa Casa de São Paulo, Departamento de Saúde Coletiva, São Paulo, SP, Brasil; Universidade Federal do Espírito Santo, Vitória, ES, Brasil; Universidade Federal do Ceará, Departamento de Saúde Comunitária, Fortaleza, CE, Brasil; Faculdade Ciências Médicas Santa Casa de São Paulo, São Paulo, SP, Brasil; Secretaria de Estado da Saúde do Amapá, Macapá, AP, Brasil; Universidade Federal de Santa Catarina, SC, Brasil; Organização Pan-Americana da Saúde, Brasília, DF, Brasil; Instituto Evandro Chagas, Belém, PA, Brasil; Faculdade de Ciências Médicas Santa Casa de São Paulo, Departamento de Saúde Coletiva, São Paulo, SP, Brasil; Universidade Federal do Rio Grande do Norte, Natal, RN, Brasil; Universidade Federal do Ceará, Departamento de Saúde Comunitária, Fortaleza, CE, Brasil; Universidade Federal de Mato Grosso, Cuiabá, MT, Brasil; Universidade Federal do Paraná, Curitiba, PR, Brasil; Universidade Federal de Goiás, Goiânia, GO, Brasil; Universidade Federal do Piauí, Teresina, PI, Brasil; Universidade de Pernambuco, Faculdade de Ciências Médicas, Pernambuco, PE, Brasil; Instituto de Saúde Coletiva, Universidade Federal da Bahia, Salvador, BA, Brasil; Secretaria de Estado da Saúde de Alagoas, Maceió, AL, Brasil; Universidade Federal do Acre, Rio Branco, AC, Brasil; Universidade Federal do Maranhão, Departamento de Saúde Pública, São Luís, MA, Brasil; Universidade Federal de Sergipe, Aracaju, SE, Brasil; Secretaria Municipal de Saúde, Boa Vista, RR, Brasil; Fundação Oswaldo Cruz, Mato Grosso do Sul, Campo Grande, MS, Brasil; Fundação Oswaldo Cruz, Escola Nacional de Saúde Pública Sergio Arouca, Rio de Janeiro, RJ, Brasil; Universidade Federal do Rio Grande do Sul, Porto Alegre, RS, Brasil; Fundação Oswaldo Cruz, Instituto de Pesquisa René Rachou, Belo Horizonte, MG, Brasil; Secretaria de Desenvolvimento Ambiental de Rondônia, Porto Velho, RO, Brasil; Universidade de Brasília, Brasília, DF, Brasil

**Keywords:** Programas de Imunização, Cobertura Vacinal, Hesitação Vacinal, Vacinas, Inquéritos Epidemiológicos

## Abstract

**Objetivo:**

Estimar a cobertura vacinal, identificar barreiras e hesitação à vacinação em crianças com até 24 meses, nascidas em 2017 e 2018, residentes na área urbana de Rio Branco-AC.

**Métodos:**

Inquérito populacional realizado de 2020 a 2021, que avaliou as características sociodemográficas e a situação vacinal em crianças nascidas entre 2017 e 2018.

**Resultados:**

Entre as 451 crianças estudadas, as coberturas vacinais foram inferiores a 80%. A menor cobertura para doses aplicadas (76,3%; IC_95_% 70,5;81,3) e oportunas (27,4%; IC_95_% 23,1;32,1) foi para o reforço da meningocócica C. As afirmações “vacinas causam reações adversas graves” (26,4%; IC_95_% 18,1;36,8) e “não precisa da vacina para doenças que não existem mais” (22%; IC_95_% 15,7;29,8) foram as mais frequentes quanto à hesitação vacinal. A falta da vacina foi a principal barreira assistencial (86,6%; IC_95_% 71,8;94,3).

**Conclusão:**

As coberturas vacinais em crianças nascidas em 2017 e 2018 ficaram abaixo da meta preconizada nos esquemas completos de doses aplicadas, válidas e oportunas.

## Contribuições do estudo

**Principais resultados:**

Coberturas inferiores a 80%. Principais barreiras: dificuldades de acesso e ausência de vacina. Acreditar que os eventos supostamente atribuíveis à vacinação são graves foi a afirmação mais frequente para justificativa da hesitação vacinal.

**Implicações para os serviços:**

É necessário evitar perda de oportunidade vacinal, com garantia de vacinas e profissionais habilitados nas unidades de saúde, além de orientar a comunidade sobre o calendário de vacinação e aprazamentos.

**Perspectivas:**

Estudos prospectivos e qualitativos auxiliam na avaliação dos fatores associados, e na compreensão das barreiras e hesitação vacinal; a avaliação de custo-efetividade de programas voltados para o papel dos profissionais de saúde é essencial.

## INTRODUÇÃO

Com a finalidade de controlar os indicadores das doenças imunopreveníveis, a Organização Mundial da Saúde (OMS) recomenda um percentual de cobertura vacinal superior a 95%, sendo esta uma relevante medida de saúde pública.^1^ Todavia, a tendência de decréscimo e desigualdades nas coberturas se evidenciam nos níveis regional e global, e países de alta renda apresentam cobertura mais elevada quando comparados com os de baixa renda.^2^ Entre 2019 e 2021, um estudo que avaliou a desigualdade global e regional em seis regiões da OMS (África, América, Mediterrâneo Oriental, Europa, Sudeste Asiático e Pacífico Ocidental) mostrou que, dessas regiões, a Europa apresentou a cobertura mais elevada para um grupo de 11 vacinas do calendário infantil, enquanto a África apresentou as menores coberturas.^3^ Outro estudo mostrou que menos da metade dos países da América Latina alcançaram a cobertura de 90%.^4^

Os desafios para melhoria da cobertura vacinal não se restringem apenas a questões econômicas. O aumento da noção de risco de eventos supostamente atribuíveis à vacinação ou imunização (ESAVI) e a diminuição da compreensão de risco das doenças também se tornam empecilhos, gerando hesitação vacinal, mesmo em países de alta renda.^5^

O Programa Nacional de Imunizações (PNI) do Brasil tem se destacado mundialmente, por coordenar ações de imunização de forma universal e gratuita.^6^ Contudo, o declínio das coberturas vacinais tem gerado preocupação quanto à possibilidade de retorno de doenças.^7^

Um estudo ecológico realizado no Brasil, com dados do Sistema de Informação do PNI (SI-PNI), entre 2011 e 2020, observou tendências decrescentes significativas na cobertura vacinal dos imunobiológicos: vacina contra tuberculose, com variação percentual média anual negativa de 3,58%; pentavalente, de 4,10%; poliomielite, de 2,76%; e tríplice viral, de 2,56%.

O Norte apresentou a maior queda na cobertura dessas vacinas entre as regiões do país. Menor desenvolvimento socioeconômico, dificuldades na disponibilidade de vacinas e barreiras geográficas e de acesso contribuíram para as disparidades.^8^

Com base nas estimativas do SI-PNI para cobertura vacinal, proporção de abandono e porte populacional, a maior concentração de municípios com risco alto e muito alto de transmissão de doenças imunopreveníveis do país estão nos estados do Acre, Amazonas, Amapá, Pará, Piauí e Roraima.^9^ O Acre, localizado no oeste amazônico, apresentou em 2021 a segunda menor cobertura para a primeira dose da vacina tríplice viral (60,2%) e a quinta menor cobertura da terceira dose contra poliomielite (61,8%).^10^

Embora a cobertura vacinal no país tenha sido predominantemente estimada a partir de dados registrados nas unidades de saúde, algumas objeções são apontadas quanto aos erros de registro e estimativas da população-alvo. Para superar tais problemas, a realização de inquéritos populacionais, com o objetivo de melhor estimar a cobertura vacinal, medir as desigualdades e investigar barreiras à vacinação, se configura como uma importante iniciativa.^11^

Assim, este estudo objetiva estimar a cobertura vacinal e identificar barreiras e hesitação à vacinação entre crianças até 24 meses, nascidas entre 2017 e 2018, e residentes na área urbana de Rio Branco-AC. 

## MÉTODOS

Trata-se de um inquérito domiciliar de base populacional, integrante do Inquérito Nacional de Cobertura Vacinal 2020 (INCV 2020), realizado de dezembro de 2020 a maio de 2021, com crianças nascidas em 2017 e 2018, residentes na área urbana de Rio Branco-AC, cuja metodologia está detalhada em outro artigo.^11^

Rio Branco é a capital do estado do Acre. Em 2020, sua população residente estimada foi de 413.418, sendo 7,53% de crianças entre 0 e 4 anos, e taxa de natalidade de 13,89 nascidos vivos por mil habitantes. A população-alvo desta pesquisa foram os 12.955 nascidos vivos em 2017 e 2018 (6.460 e 6.495, respectivamente), de mães residentes no município, segundo dados do Sistema de Informações sobre Nascidos Vivos (Sinasc).

A amostragem foi realizada em três estágios. O primeiro correspondeu à estratificação por setores censitários (A – alto, B – médio alto, C – médio baixo e D – baixo), classificados de acordo com o Censo Demográfico 2010. No segundo, procedeu-se à formação de conglomerados de setores censitários, baseada no número estimado de nascidos vivos do Sinasc (2017-2018), sendo sorteados quatro conglomerados em cada estrato socioeconômico. No terceiro, foi realizada a busca das crianças residentes nos conglomerados. Considerando uma prevalência esperada de crianças vacinadas de 70%, intervalo de confiança de 95% (IC_95_%) e efeito do desenho de 1,4, o tamanho amostral estimado foi de 452 crianças no município. 

Os dados foram coletados por uma empresa especializada. A identificação das crianças foi efetuada através dos dados do Sinasc (2017-2018). Nas situações que impediram a realização da entrevista (endereços não encontrados; crianças que não residiam no local; recusa; e responsável não estava em casa após duas tentativas), foi efetuada a substituição por outra criança nascida em 2017 ou 2018, residente no conglomerado. Os entrevistadores utilizaram um questionário padronizado (via dispositivo eletrônico), respondido pela mãe ou responsável pela criança. Informações sobre vacinas foram obtidas da caderneta de vacinação. 

A cobertura vacinal foi calculada por meio da divisão entre o número de crianças que receberam as doses das vacinas do esquema pelo total de crianças da amostra, multiplicado por 100, levando-se em consideração o calendário até 24 meses.^13^ Os esquemas foram divididos em vacinas que deveriam ser administradas de 0-12 e de 12-24 meses de vida, sendo posteriormente analisada a combinação das doses administradas de 0-24 meses de vida. Para cada esquema, foi feito o cálculo da cobertura, considerando-se três indicadores: doses aplicadas, válidas e oportunas.

O esquema de 0-12 meses inclui uma dose da vacina contra tuberculose ‒ bacilo de Calmette-Guérin (BCG) ‒, uma contra hepatite B, três doses da pentavalente, três doses contra poliomielite, duas doses e um reforço da pneumocócica, duas doses da vacina oral de rotavírus humano (VORH), duas doses e um reforço da meningocócica C, primeira dose da tríplice viral e uma contra febre amarela. O esquema de 12-24 meses inclui uma dose da vacina contra hepatite A, a segunda dose da tríplice viral, um reforço contra poliomielite, um reforço da tríplice bacteriana (DTP) e uma dose contra varicela. No esquema vacinal de 0-24 meses, ocorre a junção dos dois esquemas anteriores.^13^

No cálculo da cobertura vacinal das doses aplicadas, o numerador foi o somatório de crianças que tiveram as vacinas administradas para cada esquema (0-12, 12-24 e 0-24 meses), sem se levar em consideração a época e os intervalos; nas doses válidas e oportunas, o numerador foi o somatório das que tiveram as vacinas para cada esquema administradas em intervalos específicos.^12^ O denominador, nas três doses, foi o total de crianças da amostra.

A hesitação vacinal seguiu o conceito dos 3Cs da OMS, que se baseia em três categorias principais: confiança, complacência e conveniência.^13^ As barreiras foram relacionadas a motivos pessoais ou assistenciais que dificultaram ou impediram a vacinação. 

Foi realizada uma análise descritiva, através de frequências relativas, das variáveis sociodemográficas e econômicas: idade materna ao nascimento da criança (≤ 20 anos; 21 a 34 anos; ≥ 35 anos), raça/cor da pele materna (branca; não branca), escolaridade materna (anos de estudo: 0-8; 9-12; 13-15; ≥ 16); situação conjugal materna (tem companheiro: sim; não); número de filhos vivos (1; 2-3; ≥ 4); trabalho materno remunerado (sim; não); sexo da criança (masculino; feminino); raça/cor da pele da criança (branca; não branca); ordem de nascimento da criança (primeiro; segundo/terceiro; quarto ou mais); Bolsa Família (sim; não); e renda familiar mensal (≤ R$ 1.000,00; R$ 1.001,00 a R$ 3.000,00; > R$ 3.000,00). 

Foram coletadas informações referentes à hesitação vacinal e às principais barreiras para vacinação, a saber: “vacinas são importantes para a saúde da criança e para a proteção coletiva”; “a criança precisa tomar vacina para doenças que não existem mais”; “vacinas causam reações adversas graves”; “confiança nas vacinas distribuídas pelo governo”; “dificuldades para vacinar a criança”; “a criança já deixou de ser vacinada, mesmo sendo levada ao posto de vacinação”; e “deixou de vacinar a criança por decisão própria”. 

Em todas as análises, foram considerados os valores correspondentes ao efeito proveniente da utilização de amostra por conglomerados em múltiplos estágios, permitindo a estimação sem viés dos parâmetros de interesse na população.^11^ As estimativas consideraram os pesos correspondentes aos diferentes tamanhos de população em cada estrato. As análises foram realizadas utilizando-se o SPSS versão 21.0. 

O estudo foi aprovado pelos Comitês de Ética em Pesquisa com Seres Humanos do Instituto de Saúde Coletiva da Universidade Federal da Bahia, sob parecer n^o^ 3.366.818, em 4 de junho de 2019, com Certificado de Apresentação de Apreciação Ética (CAAE) 4306919.5.0000.5030, e da Irmandade da Santa Casa de São Paulo, sob parecer n^o^ 4.380.019, em 4 de novembro de 2020, com CAAE 39412020.0.0000.5479.

## RESULTADOS

Foram incluídas 451 crianças, com percentual de perda de 0,22%. Do total de crianças, 50% (IC_95_% 42,5;57,5) eram do sexo masculino; a maioria, não branca (73,8%; IC_95_% 67,5;80,5); e ocupavam a segunda/terceira posição na ordem de nascimento (45,6%; IC_95_% 39,4;51,9). Quanto às características familiares, a maioria das mães estava na faixa de 21 a 34 anos (63,7%; IC_95_% 57,2;69,7), era de raça/cor da pele não branca (81,5%; IC_95_% 72,3;88,1), com 13 a 15 anos de estudo (47,8%; IC_95_% 37,2;58,6), possuía companheiro (74,0%; IC_95_% 65,7;80,9), tinha entre dois e três filhos (48,6%; IC_95_% 39,4;58,0), não trabalhava (57,4%; IC_95_% 46,8;67,3), ocupava o estrato socioeconômico D (41,2%; IC_95_% 25,3;59,2), não recebia Bolsa Família (63%; IC_95_% 54,6;70,8) e possuía renda familiar mensal de R$ 1.001,00 a R$ 3.000,00 (54,2%; IC_95_% 42,9;65,1) (Tabela 1). 

**Tabela 1:** Caracterização dos aspectos sociodemográficos e econômicos em crianças até 24 meses e suas mães, residentes em Rio Branco, Acre, Inquérito de Cobertura Vacinal, 2020 (n = 451)

Variáveis	%	IC_95_ **%** ^a^
**Raça/cor da pele da criança referida pelo responsável**		
Branca	26,2	19,5;34,3
Não branca	73,8	65,7;80,5
**Ordem de nascimento da criança**		
Primeiro	41,3	33,8;49,2
Segundo/terceiro	45,6	39,4;51,9
Quarto ou mais	13,0	9,3;18,1
**Faixa etária materna ao nascimento da criança**		
≤ 20 anos	8,5	4,7;14,9
21 a 34 anos	63,7	57,2;69,7
≥ 35 anos	27,8	22,6;33,8
**Raça/cor da pele materna autorreferida** ^b^		
Branca	18,5	11,9;27,7
Não branca	81,5	72,3;88,1
**Escolaridade materna (anos de estudo)** ^b^		
0 a 8 anos	12,8	7,1;22,0
9 a 12 anos	10,6	7,1;15,5
13 a 15 anos	47,8	37,2;58,6
16 anos ou mais	28,8	23,1;35,3
**Situação conjugal materna (tem companheiro)** ^b^		
Sim	74,0	65,7;80,9
Não	26,0	19,1;34,3
**Número de filhos maternos vivos**		
1	34,6	25,5;44,9
2-3	48,6	39,4;58,0
4 ou mais	16,8	12,3;22,5
**Trabalho materno remunerado** ^b^		
Sim	42,6	32,7;53,2
Não	57,4	46,8;67,3
**Estrato socioeconômico**		
A	7,3	4,5;11,7
B	16,4	11,0;23,6
C	35,1	21,1;52,2
D	41,2	25,3;59,2
**Bolsa Família**		
Sim	37,0	29,2;45,4
Não	63,0	54,6;70,8
**Renda familiar mensal** ^b^		
≤ R$ 1.000,00	27,1	18,2;38,4
De R$ 1.001,00 a R$ 3.000,00	54,2	42,9;65,1
> R$ 3.000,00	18,7	13,3;25,5

a) IC_95_%: Intervalo de confiança de 95%; b) Variável com *missings* (< 5%).

Na administração das vacinas de 0 a 12 meses, 78,0% (IC_95_% 72,2;82,8) tiveram cobertura vacinal com doses aplicadas; 58,8% (IC_95_% 54,0;65,5), com doses válidas; e 12,4% (IC_95_% 8,6;17,7), com doses oportunas. Das vacinas de 12 a 24 meses, 64,2% (IC_95_% 57,9;70,1) das crianças tiveram cobertura vacinal com doses aplicadas; 55,5% (IC_95_% 48,9;61,9), com doses válidas; e 9,6% (IC_95_% 5,6;46,0), com doses oportunas. Das vacinas de 0 a 24 meses, 60,3% (IC_95_% 53,0;67,2) tiveram cobertura vacinal com doses aplicadas; 39,3% (IC_95_% 35,0;43,8), com doses válidas; e 5,4% (IC_95_% 2,2;12,6), com doses oportunas (Tabela 2).

**Tabela 2:** Cobertura vacinal por esquema e por vacina, segundo doses aplicadas, válidas e oportunas em crianças até 24 meses, residentes em Rio Branco, Acre, Inquérito de Cobertura Vacinal, 2020 (n = 451)

Esquemas e vacinas	Doses aplicadas % (IC_95_ **%**)^a^	Doses válidas % (IC_95_ **%**)^a^	Doses oportunas % (IC_95_ **%**)^a^
Esquema vacinal de 0-12 meses	78,0 (72,2;82,8)	58,8 (54,0;65,5)	12,4 (8,6;17,7)
Esquema vacinal de 12-24 meses	64,2 (57,9;70,1)	55,5 (48,9;61,9)	9,6 (5,6;46,0)
Esquema vacinal de 0-24 meses	60,3 (53,0;67,2)	39,3 (35,0;43,8)	5,4 (2,2;12,6)
BCG	92,3 (89,0;94,7)	92,3 (89,0;94,7)	89,4 (85,9;92,1)
Hepatite B	92,9 (89,5;95,2)	92,9 (89,5;95,2)	89,4 (85,9;92,2)
Pentavalente (1ª dose)	96,1 (91,8;98,2)	96,0 (91,8;98,1)	74,8 (69,2;79,6)
Poliomielite (1ª dose)	96,1 (91,8;98,2)	95,9 (91,5;98,0)	83,8 (79,6;87,3)
Pneumocócica (1ª dose)	95,1 (91,2;97,3)	94,6 (90,8;96,9)	81,2 (76,3;85,3)
Rotavírus (1ª dose)	91,9 (88,7;94,2)	90,3 (87,6;92,5)	76,9 (72,2;81,0)
Meningocócica C (1ª dose)	94,8 (90,9;97,1)	94,8 (90,9;97,1)	68,7 (61,6;75,1)
Pentavalente (2ª dose)	95,3 (91,5;97,4)	95,1 (91,4;97,3)	56,7 (50,3;62,9)
Poliomielite (2ª dose)	95,4 (91,6;97,5)	95,4 (91,6;97,5)	65,4 (59,1;71,3)
Pneumocócica (2ª dose)	93,6 (90,3;95,9)	93,2 (89,9;95,5)	61,4 (52,7;69,5)
Rotavírus (2ª dose)	86,8 (81,9;90,5)	69,4 (63,1;75,1)	52,9 (46,1;59,5)
Meningocócica C (2ª dose)	91,0 (87,1;93,8)	90,1 (86,1;93,1)	47,8 (41,8;53,8)
Pentavalente (3ª dose)	92,8 (89,3;95,3)	91,7 (88,3;94,1)	37,3 (28,7;46,8)
Febre amarela	89,2 (85,2;92,3)	85,0 (80,4;88,7)	39,1 (34,2;44,2)
Poliomielite (3ª dose)	91,2 (86,6;94,2)	90,9 (86,4;94,0)	50,3 (41,2;59,4)
Pneumocócica (reforço)	84,1 (79,2;87,9)	82,1 (77,1;86,2)	37,8 (31,1;45,0)
Meningocócica C (reforço)	76,3 (70,5;81,3)	73,9 (67,4;79,6)	27,4 (23,1;32,1)
Tríplice viral (1ª dose)	91,4 (88,1;93,9)	90,5 (87,8;92,7)	40,1 (34,9;45,5)
Hepatite A	89,1 (85,2;92,1)	88,1 (84,2;91,2)	42,4 (35,7;49,5)
Tríplice viral (2ª dose)	78,9 (72,8;83,9)	75,7 (69,7;80,8)	29,8 (22,8;37,9)
Poliomielite (1º reforço)	84,8 (80,9;87,9)	81,2 (75,9;85,6)	39,2 (31,2;47,8)
DTP (1º reforço)	78,2 (72,6;83,0)	78,2 (72,6;83,0)	29,7 (23,0;37,5)
Varicela	80,8 (74,4;85,9)	79,1 (73,0;84,0)	32,0 (24,5;40,6)

a) IC_95_%: Intervalo de confiança de 95%.

Ao se observarem as vacinas isoladamente, a menor cobertura para doses aplicadas se verificou no reforço da meningocócica (76,3%; IC_95_% 70,5;81,3), seguida do reforço da DTP (78,2%; IC_95_% 72,6;83,0), sendo as maiores coberturas registradas na 1ª dose da pentavalente (96,1%; IC_95_% 91,8;98,2) e na 1ª dose contra poliomielite (96,1%; IC_95_% 91,8;98,2). As menores coberturas para as doses válidas foram a da 2ª dose da VORH (69,4%; IC_95_% 63,1;75,1) e a do reforço da meningocócica C (73,9%; IC_95_% 67,4;79,6); as maiores coberturas foram a da 1ª dose da vacina pentavalente (96,0%; IC_95_% 91,8;98,1) e a da 1ª dose da vacina contra poliomielite (95,9%; IC_95_% 91,5;98,0). As menores cobertura para doses oportunas foram a do reforço da meningocócica C (27,4%; IC_95_% 23,1;32,1), do primeiro reforço da DTP (29,7%; IC_95_% 23,0;37,5) e da 2ª dose da vacina tríplice viral (29,8%; IC_95_% 22,8;37,9); as maiores coberturas foram da BCG (89,4%; IC_95_% 85,9;92,1) e da vacina contra hepatite B (89,4%; IC_95_% 85,9;92,2) (Tabela 2).

No que concerne à hesitação vacinal, a confiança foi avaliada mediante questionamentos sobre reações adversas graves e confiança nas vacinas. Na complacência, as questões tematizavama a decisão de não vacinar a criança (indivíduos que responderam “sim” falaram sobre os motivos da decisão); a importância da vacina para a saúde da criança e a proteção coletiva; e a necessidade da vacina para doenças que não existem mais. Na conveniência, o questionamento foi voltado para a dificuldade em levar a criança para vacinar (indivíduos que responderam “sim” relataram quais eram essas dificuldades). A criança não ter sido vacinada, mesmo sendo levada ao posto de vacinação, também representou uma barreira (indivíduos que responderam “sim” relataram qual foi o motivo da vacinação não ter acontecido).

Nos dados de hesitação vacinal, a maioria concordou que as vacinas são importantes para a saúde da criança (99,9%; IC_95_% 99,5;100,0) e a proteção coletiva (99,2%; IC_95_% 95,6;99,9). Porém, um percentual de 22% (IC_95_% 15,7;29,8) achou que a criança não precisa tomar vacina para doenças que não existem mais, e 26,4% (IC_95_% 18,1;36,8), que as vacinas causam reações adversas graves. A maioria (94,3%; IC_95_% 89,3;97,1) confia nas vacinas distribuídas pelo governo. Tiveram alguma dificuldade para levar a criança para vacinar 6,1% (IC_95_% 3,3;11,0), 2,7% (IC_95_% 1,3;5,7) já deixaram de vacinar a criança por decisão própria, e 40,1% (IC_95_% 33,1;47,6) já levaram a criança para vacinar e não obtiveram êxito (Tabela 3). Nas barreiras para vacinação, entre os que relataram dificuldades para levar a criança para vacinar (6%), as mais frequentes foram “o posto fica longe” (77,5%; IC_95_% 53,9;91,0), e “falta de transporte” (41,5%; IC_95_% 21,4;65,0). Nos motivos para a criança não ter sido vacinada, mesmo sendo levada ao posto (40,1%), o mais frequente foi a falta de vacina (86,6%; IC_95_% 71,8;94,3) (Tabela 4).

**Tabela 3:** Caracterização da hesitação vacinal em crianças até 24 meses, residentes em Rio Branco, Acre, Inquérito de Cobertura Vacinal, 2020 (n = 451)

Variáveis	%	IC_95_ **%** ^a^
**Vacinas são importantes para a saúde da criança**		
Desfavorável/indiferente	0,1	0,0;0,5
Favorável	99,9	99,5;100,0
**Vacinas são importantes para a proteção coletiva**		
Desfavorável/indiferente	0,8	0,1;4,4
Favorável	99,2	95,6;99,9
**A criança precisa tomar vacina para doenças que não existem mais**		
Desfavorável/indiferente	22,0	15,7;29,8
Favorável	78,0	70,2;84,3
**Vacinas não causam reações adversas graves**		
Desfavorável/indiferente	26,4	18,1;36,8
Favorável	73,6	63,2;81,9
**Confiança nas vacinas distribuídas pelo serviço público**		
Desfavorável/indiferente	5,7	2,9;10,7
Favorável	94,3	89,3;97,1
**Já teve alguma dificuldade para vacinar a criança?** ^a^		
Sim	6,1	3,3;11,0
Não	93,9	89,0;96,7
**A criança já deixou de ser vacinada, apesar de ter sido levada ao posto de vacinação?**		
Sim	40,1	33,1;47,6
Não	59,9	52,4;66,9
**Alguma vez deixou de vacinar a criança por sua decisão?** ^a^		
Sim	2,7	1,3;5,7
Não	97,3	94,3;98,7
**Motivos para ter tomado a decisão de não vacinar** ^c^		
Medo da reação das vacinas ou reações ocorridas anteriormente	61,4	22,1;89,9
Pandemia	43,0	15,3;75,9
Medo de dar injeção no filho	38,5	11,3;75,4
Acredita que as vacinas fazem mal à saúde	33,9	14,1;61,5
Notícias fizeram desistir	24,8	4,2;71,5
Amigo ou parente orientou a não vacinar	10,9	1,7;46,1
Médico orientou a não vacinar	6,1	1,3;24,8
Não acredita nas vacinas	2,5	0,3;18,4
A criança estava gripada	2,3	0,3;16,7

a) IC_95_%: Intervalo de confiança de 95%; b) Variável com *missings* (< 5%); c) Tamanho amostral total reduzido (15); *missings* do tipo “não se aplica”.

**Tabela 4:** Caracterização das barreiras para vacinação em crianças até 24 meses, residentes em Rio Branco, Acre, Inquérito de Cobertura Vacinal, 2020 (n = 451)

Principais dificuldades para levar a criança para ser vacinada^a^	%	IC_95_ **%** ^c^
O posto fica longe da residência ou do trabalho	77,5	53,9;91,0
Não tem meio de transporte para ir ao posto de vacinação	41,5	21,4;65,0
Falta de tempo para levar a criança	24,5	10,6;46,8
O horário de funcionamento do posto é inadequado	18,1	6,1;42,6
Não tem dinheiro para ir ao posto de vacinação	8,6	2,6;24,4
O patrão não libera	7,7	1,7;28,7
Dificuldade de locomoção	3,7	0,8;15,5
Não sabe quando levar a criança	3,0	0,5;16,9
**Motivos para a criança não ter sido vacinada, apesar de ser levada ao posto de saúde** ^b^		
Faltou vacina	86,6	71,8;94,3
Faltou profissional de saúde	11,3	6,7;18,5
Não era dia daquela vacina	10,0	2,4;33,6
Sala de vacina fechada	6,3	2,9;13,1
Profissional não recomendou várias vacinas no mesmo dia	5,9	2,0;16,4
Faltou material	1,7	0,4;5,9
Não vacinaram porque não tinha documento	1,0	0,2;4,3
Tinha muita gente na fila e não pôde esperar	0,1	0,0;0,8

a) Tamanho amostral total reduzido (32); b) Tamanho amostral total reduzido (170); *missings* do tipo “não se aplica”; c) IC_95_%: Intervalo de confiança de 95%.

## DISCUSSÃO

O inquérito de cobertura vacinal até 24 meses de vida, realizado entre crianças nascidas em 2017 e 2018, residentes na área urbana de Rio Branco-AC, apontou coberturas vacinais inferiores a 80% nos esquemas completos de doses aplicadas, válidas e oportunas, das vacinas que deveriam ser administradas de 0 a 12 meses, de 12 a 24 meses e de 0 a 24 meses. Acreditar que as vacinas causam reações adversas graves e que não é necessário tomar vacina para doenças que não existem mais foram as afirmações mais frequentes relacionadas à hesitação vacinal. A falta da vacina foi a principal barreira assistencial. 

Ao se avaliar a cobertura vacinal, uma etapa importante é verificá-la por doses aplicadas, válidas e oportunas, sendo que esta última categoria possibilita observar a diminuição ou o aumento da vacinação no tempo oportuno.^14^ Há evidências de que as crianças com vacinação completa apresentam uma proteção 27% maior frente ao risco de morrer, comparadas àquelas em atraso vacinal.^15^

Neste estudo, a cobertura para vacinas administradas exatamente nas idades preconizadas pelo calendário foi baixa, revelando atraso vacinal. Nas doses oportunas, todas as vacinas avaliadas ficaram abaixo de 90% de cobertura, com destaque para a meningocócica C, que apresentou baixa cobertura em todas as doses.

Um estudo ecológico descreveu a cobertura por doses aplicadas da vacina meningocócica C em crianças de até 12 meses nos estados e regiões do Brasil em 2012, dois anos após a vacina ter sido incluída no calendário nacional. As regiões Norte e Nordeste não alcançaram as recomendações de 95% de cobertura. No Norte, as coberturas para a primeira, segunda dose e reforço foram, respectivamente, de 89,9%, 84,4% e 67,0%. No Acre, essas coberturas foram, respectivamente, de 89,9%, 86,2% e 51,9%.^16^

Ainda assim, uma redução considerável no número de casos estimados de meningite meningocócica foi observada após a inclusão do programa de vacinação (2011, 2012 e 2013) em crianças menores de 1 ano (65,7%; IC_95_% 44,9;86,5%) e de 1 a 4 anos (51,8%; IC_95_% 33,0;70,6%). Nas faixas etárias de 5 a 9 anos e a partir de 10 anos, houve redução estatisticamente significativa na incidência da doença em 2013, conforme estudo que avaliou o impacto da vacinação contra esse agravo.^17^

Porém, para o controle ou eliminação de doenças imunopreveníveis, não basta alcançar altas coberturas vacinais; é preciso mantê-las, para não se comprometer o progresso obtido ao longo dos anos. Nesse sentido, deve-se alertar para o controle da poliomielite e do sarampo, pois, em relação à vacinação contra essas doenças, se observam elevadas tendências de queda na cobertura.^18^

Neste estudo, a segunda dose contra rotavírus também apresentou baixa cobertura de doses válidas. No Brasil, a VORH foi introduzida em 2006 no PNI, gerando em 2007 uma diminuição de 14% nas internações por quadros diarreicos, e uma redução média de 48% nas internações de crianças menores de 5 anos.^19^ Mas, de acordo com o SI-PNI, em 2022, a cobertura da VOHR foi de 73%, sendo que a meta para a imunidade coletiva nessa vacina é de 90%.^20^

A VORH tem uma limitação para sua administração, com preconização da faixa etária bastante rigorosa. A recomendação é de duas doses, sendo a primeira aos 2 meses (podendo ser entre 1 mês e 15 dias e 3 meses e 15 dias) e a segunda aos 4 meses (podendo ser a partir de 3 meses e 15 dias a 7 meses e 29 dias). Crianças acima de 4 meses e 15 dias que não receberam nenhuma dose não poderão iniciar o esquema a partir dessa faixa etária, pois a administração fora desses prazos pode gerar complicações em que os danos se sobrepõem aos benefícios da vacina.^21^

Outro aspecto importante da cobertura vacinal é analisar o esquema completo, em vez de apenas descrever as vacinas isoladamente, pois altas coberturas de vacinas específicas não garantem altas coberturas do esquema completo.^14^ A avaliação da cobertura vacinal de 0-12, 12-24 e 0-24 meses permite acompanhar o desenvolvimento do esquema das principais vacinas que deveriam ser aplicadas na infância, identificar quedas de cobertura vacinal e o período em que essa queda é mais frequente.^22^

Neste estudo, vacinas com esquemas multidoses apresentaram queda nos percentuais nas doses subsequentes, assim como a cobertura do esquema de 12-24 meses foi menor que a do esquema de 0-12 meses, demonstrando um atraso ou abandono vacinal após o primeiro ano de vida. Em Porto Alegre-RS, de 2015 a 2017, as vacinas multidoses tiveram uma perda anual média quatro vezes maior que o aceitável pela OMS, que recomenda perdas máximas de 5% e 25% para vacinas monodose e multidoses, respectivamente.^23^ Em Araraquara-SP, um estudo de base populacional para avaliar cobertura vacinal oportuna de crianças de 12-24 meses, nascidas entre 1998 e 2013, mostrou que os atrasos são acentuados a partir dos 6 meses, estando mais relacionados à idade do que ao número de doses do esquema.^24^

Assim, a identificação dos motivos que levam o responsável pela criança a atrasar a vacina ou decidir não vacinar a criança assume grande relevância. Esse atraso pode ser influenciado tanto pelas dificuldades individuais enfrentadas pelo responsável como por crenças ou fatores relacionados aos serviços de saúde.^25^

Em pesquisa domiciliar formulada pela rede Avaaz e a Sociedade Brasileira de Imunizações (SBIm) para avaliar a percepção dos brasileiros sobre vacinas, observou-se, entre os motivos mais frequentes da hesitação, o medo dos efeitos colaterais (24,0%) e o medo de contrair a doença que se estava tentando prevenir (18,0%).^26^

Porém, é importante ressaltar que as vacinas fornecidas pelo SUS são criteriosamente analisadas, e a frequência de ESAVI grave é considerada rara, em contraste com o risco de adoecimento, sequela e mortes decorrentes de doenças imunopreveníveis.^27^

A frequência de poliomielite associada ao vírus vacinal (PAVV), por exemplo, é de 1 caso para cada 2,4 milhões de doses a 1 caso por 13 milhões de doses. Eventos como reações imunoalergênicas e anafilaxia são considerados raros (0,1%) ou extremamente raros (< 0,01%) para as vacinas do calendário infantil.^28^

Quando exploramos as barreiras para a vacinação relatadas pelos responsáveis, a distância do posto de saúde foi o fator mais frequente. A longa distância entre a residência da criança e a unidade básica de saúde (UBS) é um fator limitante que pode gerar atraso ou abandono vacinal. Um inquérito realizado na cidade de Assis Brasil-AC apontou que crianças residentes em locais mais distantes da UBS tiveram menor chance de ser vacinadas, tendo em vista que o custo do transporte para o serviço se configurou em barreira, especialmente para famílias de menor renda.^29^

As faltas de vacina e de profissional também representam barreiras para melhoria da cobertura vacinal. Ainda que se aumente o número de UBS, otimizando sua distribuição no território, a ineficiência do processo logístico de fornecimento e conservação de vacinas e a indisponibilidade de profissionais qualificados poderiam invalidar os esforços empreendidos para melhora da situação vacinal das crianças residentes em áreas mais remotas. Um fator primordial, no contexto de vacinação, é a organização dos serviços para que não faltem vacinas, profissionais e materiais para imunizar.^9^

Em Cuiabá-MT, a falta de vacina também foi o motivo mais frequente para a não vacinação (50,0%). Trata-se de uma barreira relacionada à organização dos serviços de saúde e à gestão governamental, que pode comprometer a adesão e a oportunidade de vacinação.^30^

Este foi um dos primeiros estudos de base populacional a avaliar a cobertura vacinal, abordando hesitação e barreiras para não vacinação em Rio Branco-AC. O tamanho amostral para identificar os motivos referidos pelos responsáveis para não vacinar a criança foi uma limitação, pois apresentou um alto percentual de “não se aplica”, tendo em vista que apenas 2,7% dos indivíduos relataram ter alguma dificuldade para vacinar a criança. Assim, para não se gerar a errônea ideia de estimação de probabilidade, esses motivos não foram aprofundados, sendo apenas descritos em tabela.

Embora o plano de seleção da amostra tenha sido por substituição por outra criança do mesmo conglomerado, neste estudo a perda por recusa foi de 0,22%. Assim, apesar de os inquéritos estarem sujeitos a vieses de seleção por recusas em participar, o percentual de perda por recusas foi muito baixo, reduzindo a probabilidade de viés de seleção e garantindo a representatividade da população na amostra. 

Outra limitação do estudo refere-se a um viés de informação (mais especificamente, viés de prevaricação) com relação à temática de hesitação vacinal. Por se tratar de um tema sensível, o indivíduo pode não se sentir confortável para expressar sua real opinião. Este estudo também não permitiu um olhar longitudinal e prospectivo para melhor avaliação do indicador de cobertura vacinal no município. Porém, dados relativos às vacinas foram validados a partir da caderneta de vacinação, que apresenta informações acuradas. 

O estudo apontou que os esquemas de cobertura vacinal em Rio Branco-AC estão abaixo do recomendado, com percentuais ainda menores das vacinas após o primeiro ano de vida. Além disso, mostrou que o medo dos efeitos adversos das vacinas foi o principal fator para a hesitação vacinal, enquanto a ausência de vacinas e/ou profissionais foram as principais barreiras para não vacinação, apesar de a criança ser levada à UBS. Para ampliação da cobertura vacinal, é fundamental fortalecer a atenção básica – a fim de que não ocorra perda de oportunidade vacinal por motivos gerenciais e de acesso –, promover pessoal habilitado para imunizar, garantir adequado registro vacinal e orientar a comunidade sobre a vacinação e os prazos recomendados.
